# Comparison of continuous eversion and inverting subepithelial suture in transverse preputial island flap urethroplasty in proximal hypospadias repair: A retrospective study

**DOI:** 10.1590/S1677-5538.IBJU.2019.0484

**Published:** 2020-07-31

**Authors:** Wenwen Han, Weiping Zhang, Ning Sun, Yanfang Yang

**Affiliations:** 1 Beijing Children’s Hospital Affiliated to Capital Medical University Department of Urology Beijing China Department of Urology, Beijing Children’s Hospital Affiliated to Capital Medical University, Beijing, China; 2 Children’s hospital Affiliated to Zhengzhou University Department of Urology Henan China Department of Urology, Children’s hospital Affiliated to Zhengzhou University, Henan, China

**Keywords:** Surgical Flaps, Hypospadias, Wound Healing

## Abstract

**Introduction:**

Transverse preputial island flap urethroplasty (TPIFU) is one of the most frequently performed technique for single-stage repair in proximal hypospadias. It was reported that the subepithelial urethroplasty would obviously decrease urethrocutaneous fistula (UF) complication after proximal TIP. But in the process of TPIFU, it had not been reported yet.

**Objective:**

We reviewed our experience to evaluate and compare the effect of continuous eversion suture (CES) versus continuous inversion subepithelial suture (CIS) on complication rates in the TPIFU.

**Material and methods:**

A retrospective review of all patients operated with CES and CIS in our institution between January 2017 and Jun 2017 was performed.

**Results:**

A total of 161 patients were enrolled in the research. Patients were followed up for 12~17 months. Total success rate was 73.9% (119/161). No statistically difference was found between the two groups with regard to age of patients (P=0.097), catheter size (P=0.52), time of catheterization (P=0.47), length of neourethra (P=0.20), non-urethral comorbidity (P=0.44) and post-operative infection (P=1.0). The overall postoperative complications had no statistically difference between the two groups (P=0.067). There were no statistically significant differences in the incidence of urethra-cutaneous fistula (UF) (OR=0.07, 95% CI: -0.24~0.037, P=0.22), urethral diverticulum (UD) (OR=0.026, 95% CI: -0.16~-0.056, P=0.323), urethral stricture (US) (OR=0.081, 95% CI: -0.15~0.15, P=1.0) and breakdown of urethral repair (BU) (OR=0.02, 95% CI: -0.118~-0.044, P=1.0).

**Discussion:**

The comparison of two group’s postoperative complications was feasible because there were no statistically differences among perioperative variables. It seemed as if continuous inversion subepithelial suture would promote healing. However, it indicated that the overall success rate and the incidences of UF, UD, US and BU complications had no statistically difference between groups. It might be accounted for the subtle differences of techniques changing the process of establishing prime and side branches vascularization.

**Conclusions:**

The CIS technique had no significantly different effect on the four complications rates when compared with CES in TPIFU. Thus, CES and CIS could be randomly adopted in TPIFU as personal preference.

## INTRODUCTION

Proximal hypospadias remains one of the most challenging conditions for surgical correction and transverse preputial island flap urethroplasty (TPIFU) is one of the most frequently performed technique for single-stage repair (1, 2). But urethra-cutaneous fistula (UF), urethral diverticulum (UD), urethral stricture (US) and breakdown of urethral repair (BU) are the most common postoperative complications and the complication rates range from 14.6% to 37.9% (2). Many minimal modifications of TPIFU were invented to decrease the complications (3–5). However, the exact roles of these modifications in the successful outcome of hypospadias repair are yet to be determined. It was reported that the subepithelial urethroplasty would obviously decrease UF complication after proximal TIP (6), but in the process of TPIFU, whether it is effective to decrease surgical complications had not been reported before. In order to identify a better method, we reviewed our experience to evaluate and compare the effect of continuous eversion suture (CES) versus continuous inversion subepithelial suture (CIS) on complication rates in TPIFU.

## MATERIALS AND METHODS

Patients primarily submitted to TPIFU in our department between January 1, 2017 and Jun 1, 2017 were retrospectively reviewed. Hypospadias performed by other techniques, preoperative testosterone injections and coverage of tunica vaginalis were all excluded. There was only one surgeon performing CIS, and others did CES in our hospital. All the patients were treated with standard TPIFU technique: A circumferential incision was made proximal to the corona and reached the depth of Buck’s fascia. The dorsal skin was degloved toward the proximal penis. The urethral plate was transected to correct accompanying chordee completely, while if not, dorsal plication was performed. The meatus was dropped back to the proximal penis or the penoscrotal junction. The distance between the retracted meatus and the glans tip was measured to confirm the expected length of the neourethra. The rectangular flap was outlined at the inner aspect of the dorsal prepuce according to the length of the defect. The outlined foreskin was incised and rolled into a tube over a catheter and sutured with 6-0 absorbed PDS. The size of the catheter was chosen depended on the diameter of the patient’s urethra and ranged between 6-Fr and 8-Fr. The tubularized neourethra was transposed ventrally through the glans channel and anastomosed with the native urethra with CIS or CES (according with the assigned group). The glans was incised deeply, the neourethra was placed and the new meatus was sutured on the top of the glans. The relaxed vascularized and de-epithelialized tissue was dissected to cover the neourethra. Finally, the foreskin was sutured together to cover all the penis.

**Figure 1 –:**
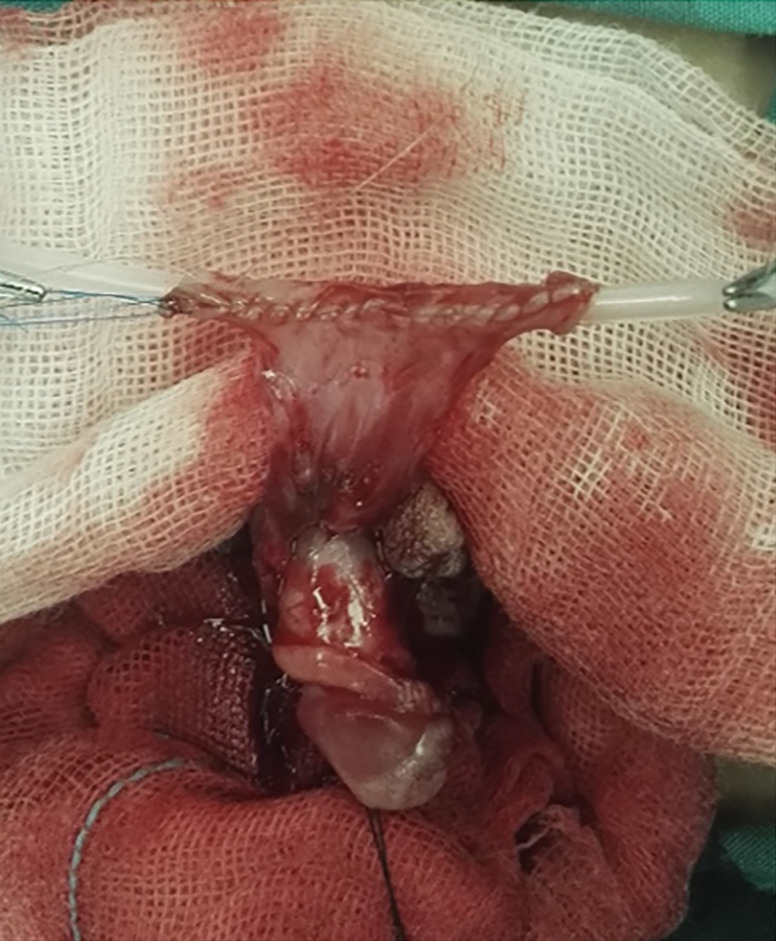
The tubularized neourethra was formed with continuous eversion suture and the anastomosing surface was rough.

**Figure 2 –:**
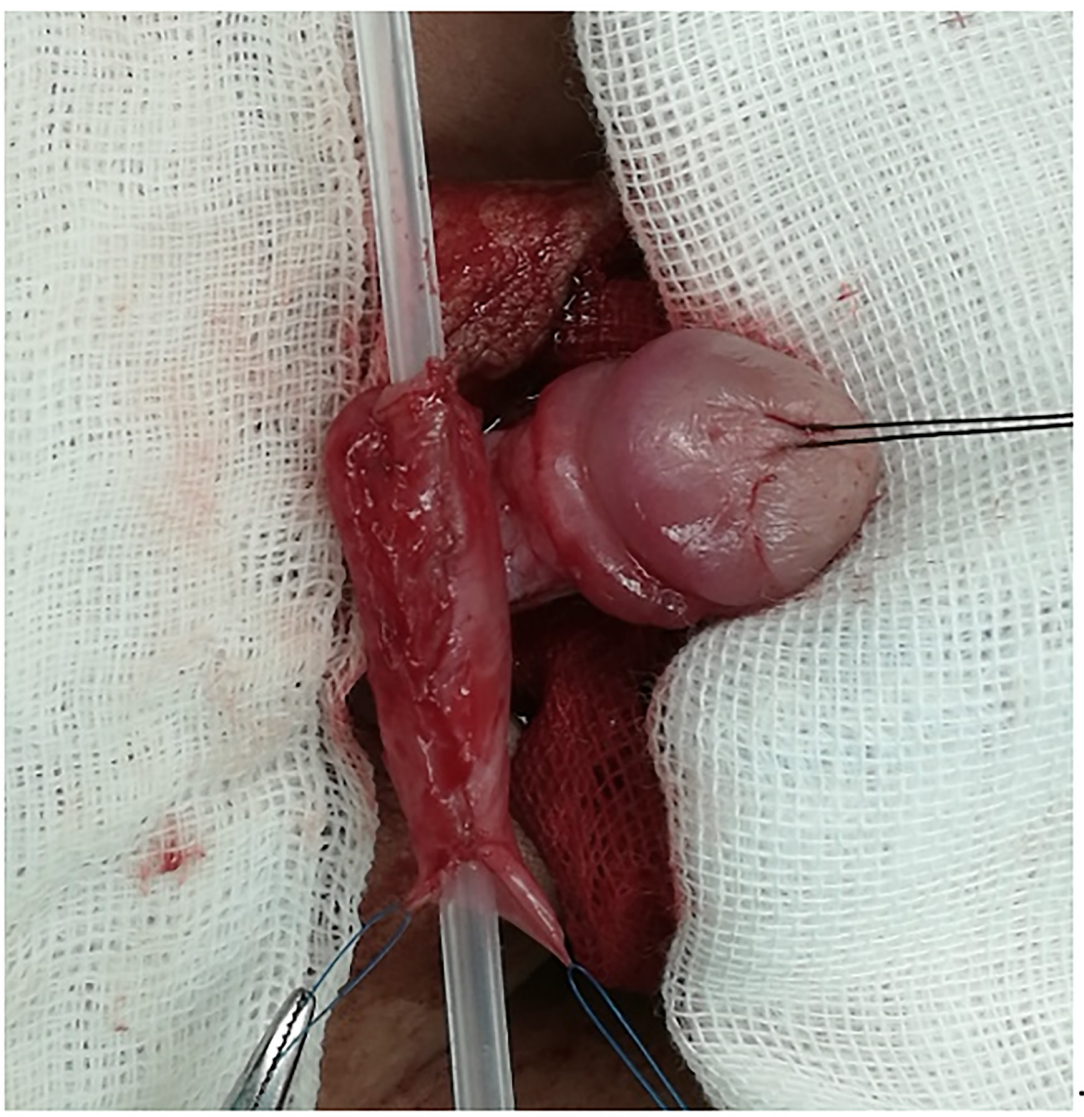
The tubularized neourethra was formed with continuous inversion subepithelial suture and the anastomosing surface was smooth.

We divided the patients into two groups according to the suture methods of the tubularized neourethra (continuous eversion suture group, Figure-1; group continuous inversion subepithelial suture group, Figure-2). All the urethral catheter used for drainage was kept for 2~6 weeks postoperatively to prevent stenosis. Venous antibiotics were applied for 3 to 5 days, oral antibiotics were continuously applied 1 week afterwards. Perioperative variables including the age of patients, catheter size, time of catheterization, length of neourethra, non-urethral comorbidity (yes or no) and postoperative infection (yes or no) were analyzed. The patients were followed-up for at least 6 months and postoperative complications of UF, UD, US and BU were recorded and analyzed. Surgical success was defined as no occurrence of these complications. The comparison between groups were analyzed using Chi-squared test and t test. All statistical calculations were performed by using SPSS 19.0. All tests were two-sided and P values <0.05 were considered significant. The research protocol was reviewed and approved by the Institutional Ethics Committee.

## RESULTS

One hundred and seventy-five cases of proximal hypospadias operated with TPIFU were identified, fourteen were excluded as not meeting the screening criteria or lost to follow-up. Since there were several surgeons performing CES but only one surgeon CIS, the number between two groups were quite imbalanced. Finally, 161 patients were included and divided into two groups: 116 in CES group and 45 in CIS group. Age ranged from 1.17 to 15.5 years, with a mean of 5.8 and 5.1 years, respectively. Patients were followed for 12~17 months (mean 13.7 months). The total success rate was 73.9% (119/161). The success rate of CES group was 69.8% (81/116), and CIS group 84.4% (38/45). Non-urethral comorbidity were hernia, cryptorchid, hydrocele, penile-scrotal transposition and cardiac anomalies.

In univariable analysis, no statistically differences were found between the two groups with regard to the age of patients (P=0.097), catheter size (P=0.52), time of catheterization (P=0.47), length of neourethra (P=0.20), non-urethral comorbidity (P=0.44), and postoperative infection (P=1.0). There were no statistically differences among perioperative variables, so the imbalanced numbers between the two groups had little effect on the following results. Chi-squared test was used to compare the incidences of postoperative complications between the two groups. The overall postoperative complications had no statistical difference between the two groups (P=0.067). There were no statistically significant differences in the incidence of UF (OR=0.07, 95% CI: -0.24~0.037, P=0.22), UD (OR=0.026, 95% CI: -0.16~-0.056, P=0.323), US (OR=0.081, 95% CI: -0.15~0.15, P=1.0) and BU (OR=0.02, 95% CI: -0.118~-0.044, P=1.0). All the data and results of statistical analysis are shown in Tables 1 and 2.

**Table 1:** Characteristics of the CES and CIS groups and P values between them.

Risk factors	Range	CES	CIS	P
Age	1.17y~15.5y	5.87±3.28	5.13±3.03	0.097
	6F	24	11	
Catheter size				0.52
	8F	92	34	
Time of catheterization	2~6 w	4.15 ±0.91	3.74±0.63	0.47
Length of neourethra	1.5~8cm	3.73±1.34	3.92±1.13	0.20
	No	102	37	
Non-urethral comorbidity				0.44
	Yes	14	8	
	No	81	38	
Complications				0.067
	Yes	35	7	
	UF	21	4	
	UD	5	0	
The four complications				0.55
	US	8	3	
	BU	1	0	
	No	106	41	
Infection				1.0
	Yes	10	4	

**CES** = continuous eversion suture; **CIS** = continuous inversion subepithelial; **UF** = cutaneous fistula; **UD** = urethral diverticulum; **US** = urethral stricture and **BU** = breakdown of urethral repair

**Table 2:** Chi-squared test among complications.

Complication		CES	CIS	P	OR	95% CI
**UF**						
	No	95	41	0.22	0.07	-0.24~0.037
	Yes	21	4			
**UD**						
	No	111	45	0.323	0.026	-0.16—0.056
	Yes	5	0			
**US**						
	No	108	42	1.0	0.081	-0.15~0.15
	Yes	8	3			
**BU**						
	No	114	45	1.0	0.02	-0.118—0.044
	Yes	2	0			

**CES** = continuous eversion suture; **CIS** = continuous inversion subepithelial; **UF** = cutaneous fistula; **UD** = urethral diverticulum; **US** = urethral stricture and **BU** = breakdown of urethral repair

## DISCUSSION

The TPIFU was first described by Duckett in 1980 (7), it has been proven to be an efficient one-stage urethroplasty to correct proximal and severe chordee hypospadias (8). Although surgeons had been making various efforts or modifications to optimize the procedure, there are still certain complications and the best options for less complications are still debated. The purpose of all the minimal modifications was to provide a tension-free, well-vascularized tubularized neourethra and improve postoperative wound healing (2). The existing modifications were only designed to improve external condition, such as soft tissue interposition, removal, increase length and width of rectangular flap and in situ tubularization of the transverse preputial island flap (3, 9). Previous studies only mentioned that a well-vascularized neourethra and preputial flaps used for repair were exceptionally important for a successful outcome (10), but neglected how to improve intrinsic element of neourethra. We derived CIS from gastrointestinal anastomosis and hypospadias TIP technique which was reported to be easier to heal than other techniques (6, 11). In this study, we described the minimal modified repair of CIS with tubularized neourethra and compared it with CES.

UF can occur inside the neourethra or at its junction to the native urethra in TPIFU. There were various causes of failure, such as overlapping sutures, distal obstruction, ischemia tissue and single-layer coverage (12). Snodgrass reported that subepithelial urethroplasty would obviously decrease UF complication after proximal TIP, but he did not explain the reason (6). For the TPIFU, the comparison between CES and CIS had not been reported before. In our study, we adopted the CIS technique in neourethra and compared them with CES. The success rate of CES group was 69.8%, and CIS 84.4%. In spite of wide discrepancy in the patient’s number of the two groups (116: 45), there were no statistical differences among perioperative variables. It demonstrated that the comparison of two group’s postoperative complications was feasible and could not be influenced by these variables. By comparing the two groups, we found that the overall success rate and the incidences of the four complications had no statistical difference.

How to explain this pathophysiology? The answer should be seek for in wound healing process. Wounds normally heal in an orderly and efficient manner characterized by overlapping phases that include inflammation, epithelialization, fibroplasia, and maturation (13). The surface of the incision abutted closer in the CIS, while the epithelium abutted closer in the CES. The basal cell proliferation and epithelial cell migration occurring in the sutured margin might be slower within the latter suture. After being anastomosed with the native urethra and glans, the neourethra would touch the anastomosing surface to the cavernosa ventrally. The external anastomosing sur-face was much smoother with CIS as shown in Figure-2. So, they could abut together more tightly. As described above, it seemed like that continuous inversion subepithelial suture was easier to heal. But the UF and BU incidences had no difference between the two groups, it might be accounted for the subtle differences of techniques changing the process of establishing prime and side branches vascularization.

There were various risk factors leading to UD and US, such as ischemia and the appearance of neourethra (14). The neourethra’s medial surface was rough with inversion incision as we all know and it might affect the results. But there were still no statistically significant differences between the two groups. It might be accounted for long time of catheterization (2~6 weeks). Someone reported that long time will lead to ischemia and infection (15), but others stated that a stent did not affect postoperative recovery (1). In our research, we did find obvious side effects, and the total success rate was acceptable (73.9%) as reported by other studies (6, 14).

The maneuvers of CES and CIS had no significantly different effect on the complications rates in TPIFU. Thus, CES and CIS could be randomly adopted in TPIFU as a personal preference.

Our study has some limitations: it had been reported that the vascular branch in the preputial island flap was associated with results of hypospadias repair (13). It’s a flaw that we did not research it in our article. We did not practice routine uroflowmetry in the research. Besides, limited number of patients and a relatively short follow--up period to observe the outcomes and complications were also limitations.
